# Laryngeal Preservation in Extraosseous Ewing's Sarcoma of the Larynx: A Case Report and Review of the Literature

**DOI:** 10.7759/cureus.105883

**Published:** 2026-03-26

**Authors:** Antaryami Sahoo, Souvik Mondal, Pritinanda Mishra, Santosh Kumar Swain, Sandip Kumar Barik

**Affiliations:** 1 Radiation Oncology, All India Institute of Medical Sciences, Bhubaneswar, Bhubaneswar, IND; 2 Pathology, All India Institute of Medical Sciences, Bhubaneswar, Bhubaneswar, IND; 3 Ear, Nose, and Throat (ENT), All India Institute of Medical Sciences, Bhubaneswar, Bhubaneswar, IND

**Keywords:** ewing's sacroma larynx, extra-osseous ewing's sarcoma, head-neck radiotherapy, laryngeal preservation, pediatric ewing's sarcoma

## Abstract

Extraosseous Ewing’s sarcoma (EES) of the larynx is an exceptionally rare malignancy, with very few cases reported in the literature. Its clinical behavior and optimal management remain poorly defined. We report the case of a 14-year-old patient who presented with hoarseness of voice. Imaging and histopathological evaluation confirmed a diagnosis of laryngeal EES. The patient was treated with systemic chemotherapy followed by radiation therapy, resulting in a favorable clinical and radiological response.

EES can occur across a broad age range and may demonstrate aggressive local growth and early metastatic potential. Due to the rarity of laryngeal involvement, standardized treatment guidelines are lacking, and management is often extrapolated from Ewing’s sarcoma at other sites. This case highlights the aggressive nature of laryngeal EES and underscores the importance of early diagnosis, comprehensive metastatic workup, and a multidisciplinary treatment approach. Additional case reports are needed to define the prognosis better and optimize management strategies.

## Introduction

Ewing's sarcoma (ES) of bone (ESB) and soft tissue presents as highly malignant small, round, blue cells, occurring more commonly in pediatric and adolescent patients. A distinctive genetic characteristic of these malignant cells, and a key feature for diagnosis, is a translocation involving the ES breakpoint region 1 (EWSR1) gene located on chromosome 22q12 [1]. Extraosseous ES (EES) is a rare, poorly differentiated tumor belonging to the ES family of tumors (ESFT). EES rarely presents in the head and neck, comprising only 8.5% to 12% of all EES tumors [2].

Laryngeal cancer is a malignant proliferation of the larynx. It usually presents as hoarseness of voice, dysphagia, and difficulty breathing, with or without a mass in the neck region. Sarcomas of the larynx constitute a rare subset of laryngeal cancers, typically accounting for less than 1% of the total cases. It rarely manifests as ES; hence, the number of documented cases for this sarcoma is not widely published in the literature [3].

The prognosis and outcome of EES are generally better than those of other anatomical locations. Important factors to consider include the patient's age and the stage of the disease at diagnosis [2].

We present a case of a pediatric patient diagnosed with ES of the larynx. Pediatric laryngeal ES is a rarely documented entity. Therefore, this report discusses this rare case and includes a review of the relevant literature.

## Case presentation

**Figure 1:**
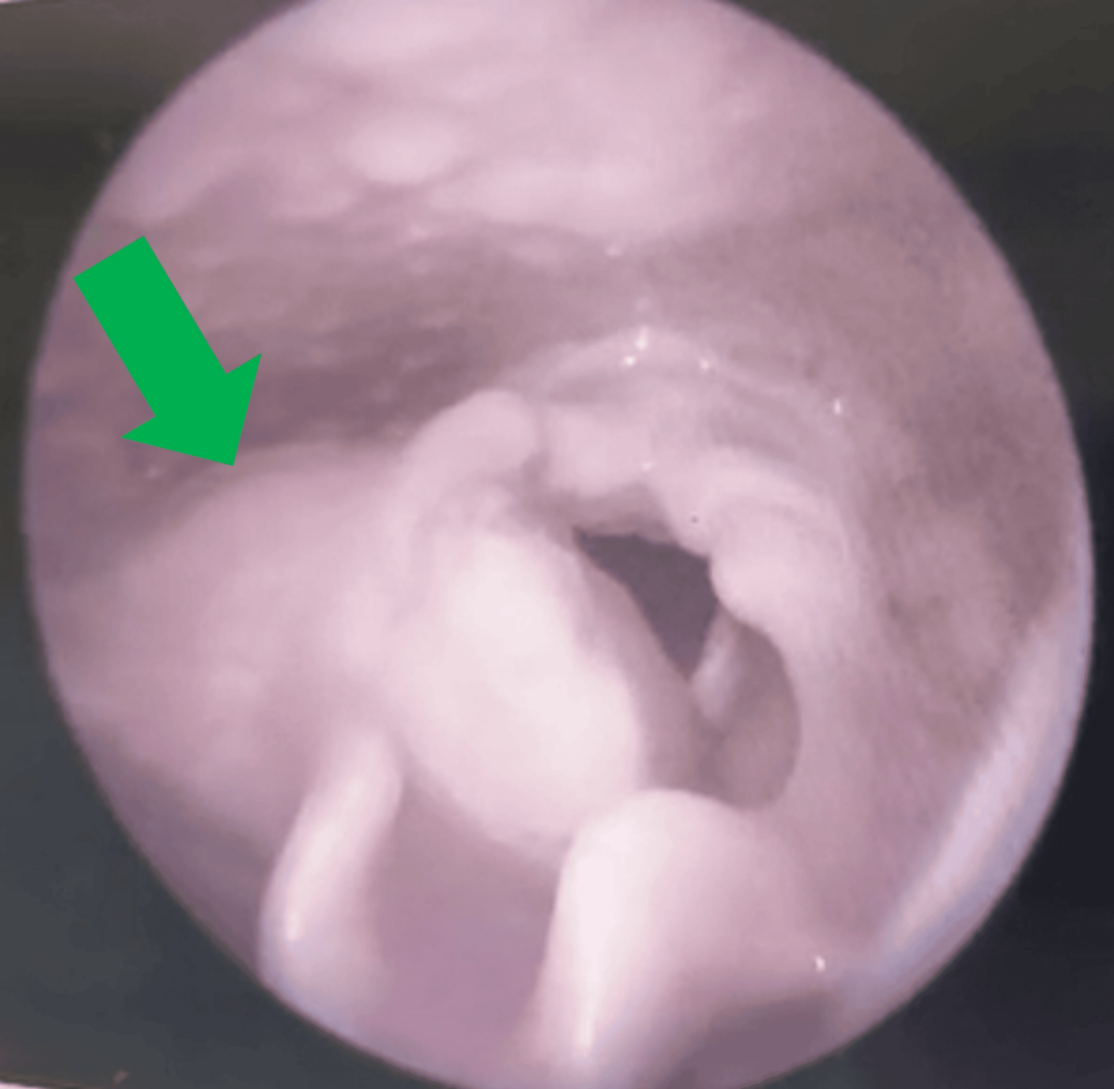
Green arrow showing large growth in FOL on the right side of the larynx involving the false vocal cord, extending into the parapharyngeal space. FOL: fiber optic laryngoscopy

**Figure 2:**
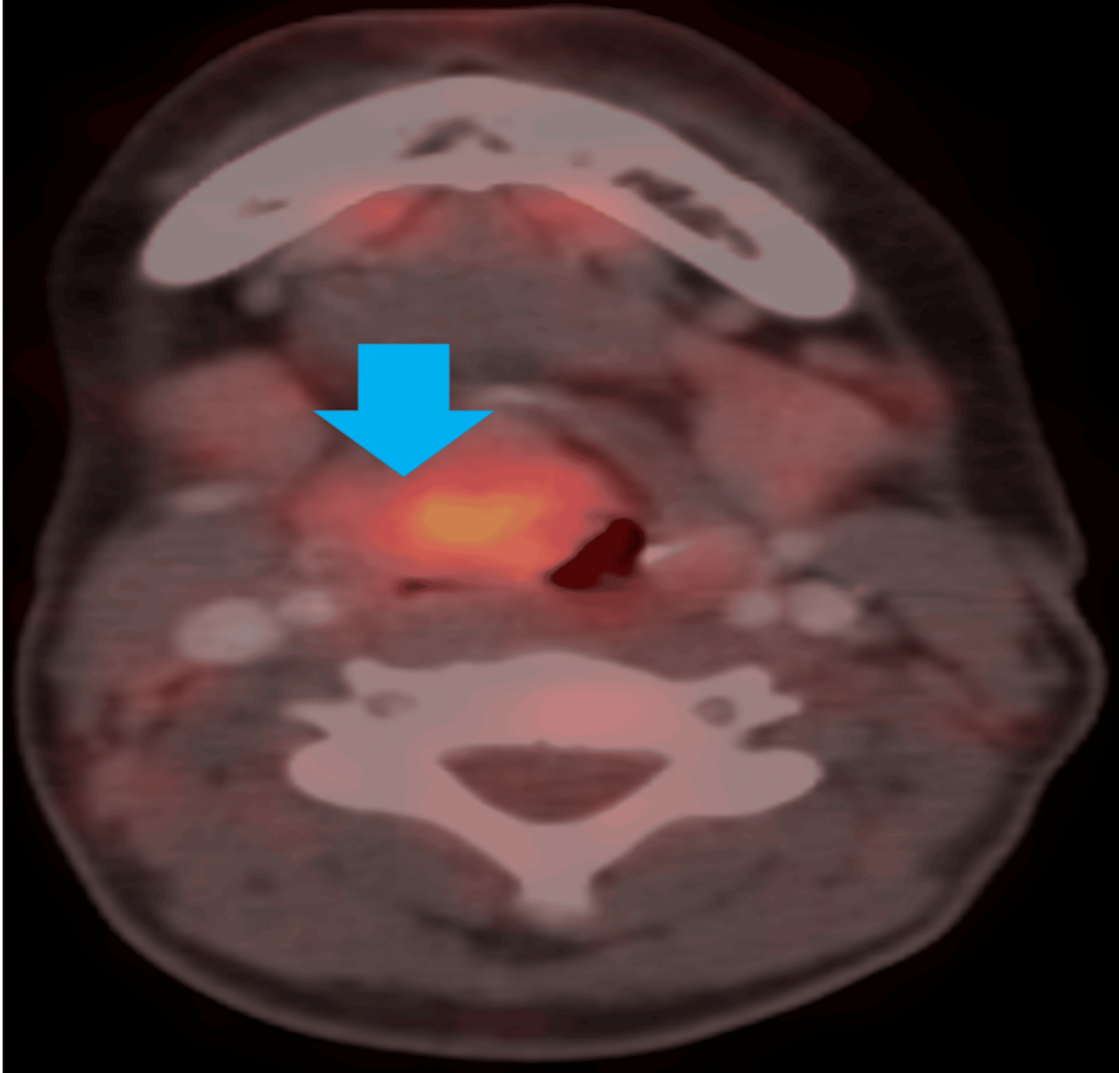
Blue arrow showing large growth in the right side of the larynx involving the vocal cord in the FDG PET/CT scan, size around 2.9 × 2.5 × 2.4 cm, SUVmax: 4.06. SUVmax: maximum standard uptake value; FDG PET/CT: fluorodeoxyglucose positron emission tomography/computed tomography

A 14-year-old female patient came with a three-month complaint of voice hoarseness. She had no associated dyspnea or dysphagia. During this time, the patient remained untreated. There was no history of tumors or other significant diseases reported in the patient's family. No significant abnormalities were noted on physical examination. Fiber optic laryngoscopy (FOL) detected a soft-tissue mass in the right false vocal cord and right aryepiglottic fold, extending to the right para-glottic space (Figure 1). A head and neck computed tomography (CT) revealed a heterogeneously minimally enhancing mass in the right supraglottic area (2.6 x 2.4 x 2.4 cm) with bilateral subcentimetric reactive lymph nodes. Laryngeal cancer was suspected based on these findings. To evaluate for distant metastases, the patient underwent a fluorodeoxyglucose (18F-FDG) positron emission tomography (PET)/CT examination. The PET/CT revealed increased FDG uptake (maximum standard uptake value (SUVmax): 4.06) in the corresponding lesions, which measured 2.9 x 2.5 x 2.4 cm and involved the right pyriform fossa, right aryepiglottic fold, and right paraglottic region (Figure 2).

**Figure 3:**
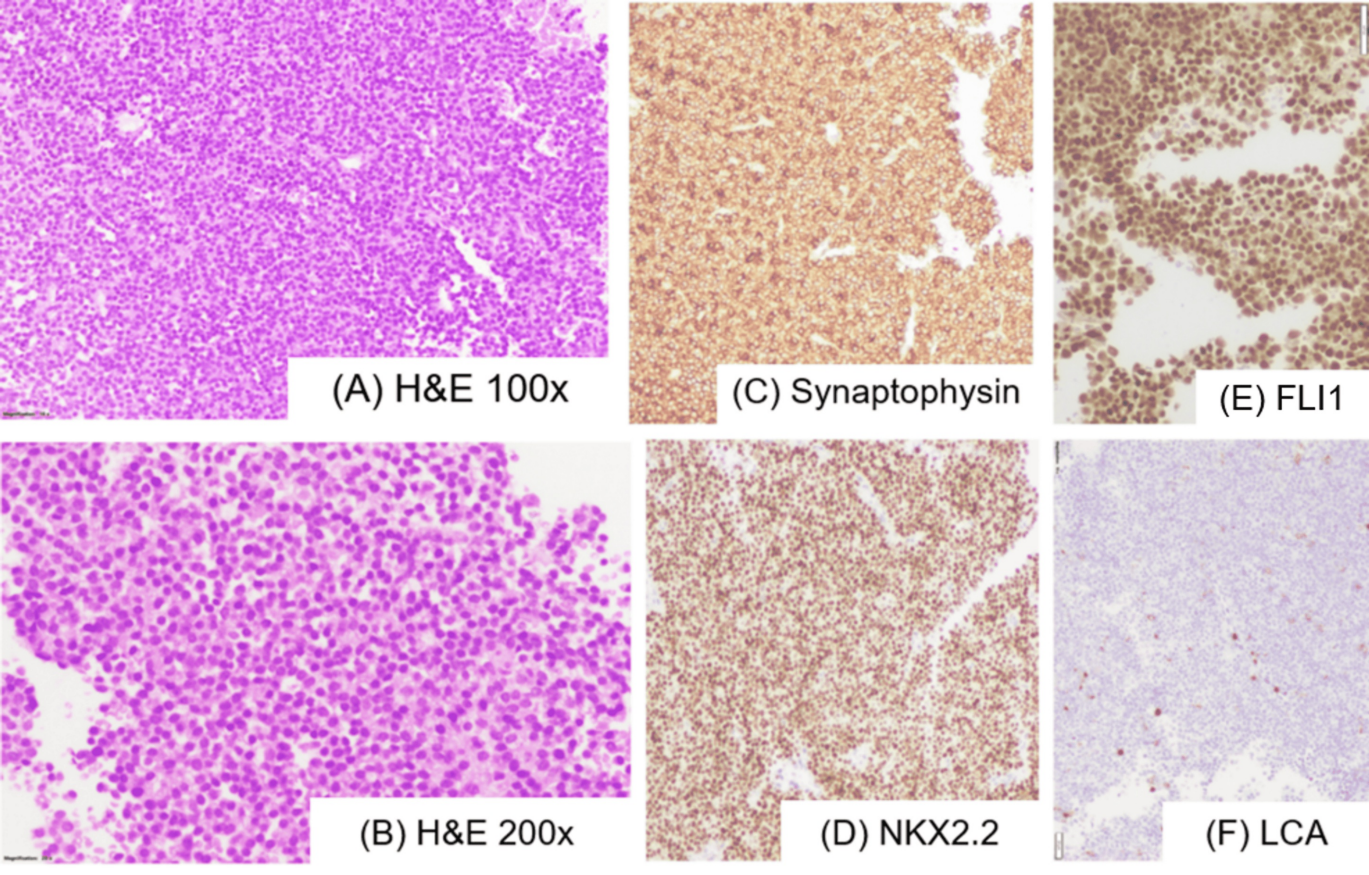
Histopathology and immunohistochemistry images showing (A) monomorphic small, round, blue cells in rosettes. (B) Cells are round with scant vacuolated cytoplasm, granular chromatin, and inconspicuous nucleoli. Tumor cells are immunopositive for synaptophysin (C), NKX2.2 (D), and FLI1 (E) and negative for LCA (F). FLI1: friend leukemia integration 1; LCA: leukocyte common antigen; H&E: hematoxylin and eosin

A tissue diagnosis was obtained via biopsy of the soft-tissue laryngeal mass, which was performed under FOL guidance. Histopathological examination using hematoxylin and eosin (H&E) staining revealed a tumor composed of small, dark, round, blue cells (Figures 3A, 3B). Immunohistochemical (IHC) analysis showed the tumor cells expressed CD99, NKX2.2, and friend leukemia integration 1 (FLI1), while staining negative for leukocyte common antigen (LCA) (Figures 3C-3F).

**Figure 4:**
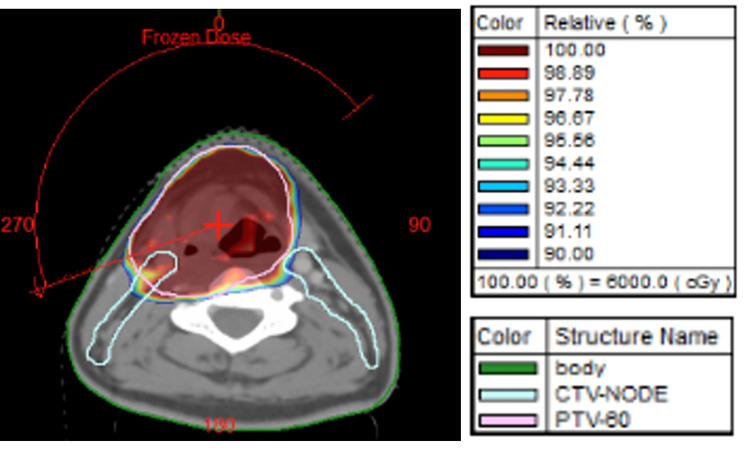
CT image showing color wash with red and blue of dose painting in the VMAT technique. Red showing 100% of dose (60 Gy), blue outside PTV 90% isodose curve. PTV: planning target volume; CT: computed tomography; VMAT: volumetric arc therapy

The final diagnosis, based on the pathology and IHC results, was ES of the larynx. The patient was planned for induction chemotherapy with an alternating vincristine (2 mg/m^2^), Adriamycin (75 mg/m^2^), cyclophosphamide (1,200 mg/m^2^), etoposide (100 mg/m^2^), and ifosfamide (1,800 mg/m^2^) (VAC/IE) regimen every three weeks for nine weeks, followed by local therapy with definitive radiotherapy and larynx preservation with growth factor support. After three cycles of chemotherapy, assessment showed partial response to treatment; then, the patient received radiotherapy with a concurrent alternate weekly V/VC regimen. The radiation dose was 60 Gy/30 fractions, followed by a sequential boost of 6 Gy/3 fractions to the gross tumor only by the volumetric arc therapy (VMAT) technique (Figure 4), along with concurrent chemotherapy. Gross disease was contoured as per FOL and CT scan findings; then, a margin of 1 cm was given for the clinical target volume (CTV), followed by a 5 mm setup margin for the planning target volume (PTV). For the boost, a 5 mm PTV margin was given from the gross tumor volume (GTV).

**Figure 5:**
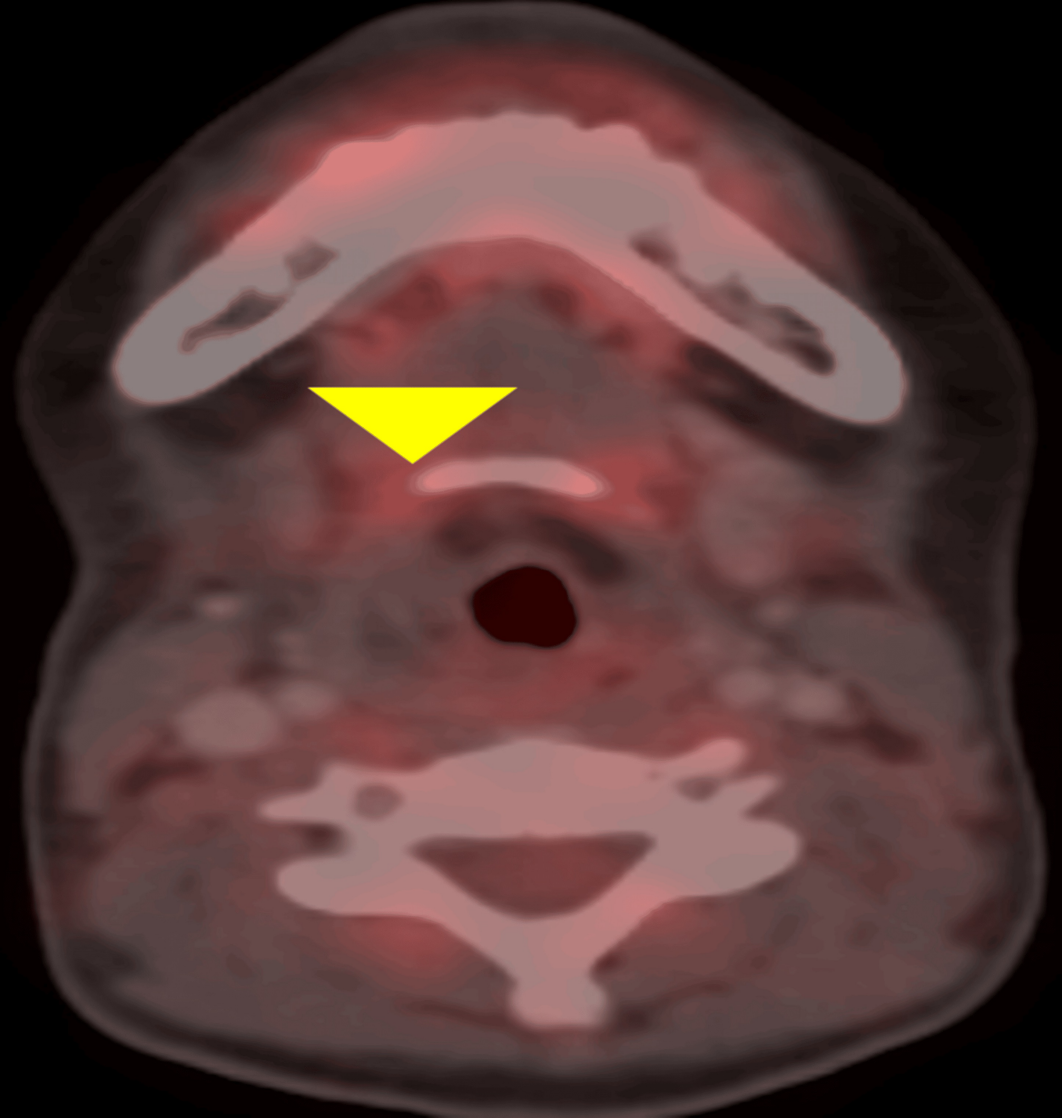
Yellow arrowhead showing the previous growth site; now, no growth in the PET/CT image post chemotherapy and post radiotherapy; complete metabolic resolution. PET/CT: positron emission tomography/computed tomography

During radiotherapy, there were manageable skin, mucosal, and hematological toxicities. All toxicities were managed with supportive care and nutrition. Post local therapy, we continued with consolidative chemotherapy till the 17th cycle; post six cycles of chemotherapy, assessment PET/CT showed complete metabolic response to the treatment (Figure 5). FOL was done again, and there was no growth visible with normal vocal cord movement (Video 1). Dysphonia resolved completely, and the patient could express all emotions with her own voice. After 10 cycles of chemotherapy, she received a total of 500 mg of Adriamycin; we kept a cumulative dose limit for Adriamycin of 400 mg/m^2^; so then, actinomycin D (1.6 mg) started from the 11th cycle instead of Adriamycin. She is continuing adjuvant chemotherapy and tolerating it well and is having a good quality of life.

**Video 1:** FOL after chemotherapy and radiotherapy, complete resolution of the tumor. FOL: fiber optic laryngoscopy

## Discussion

The ESFT encompasses classic ESB, which is the second most frequent primary bone cancer in children. EES was first identified and reported in 1969; however, it remains a rare and poorly documented pathology in medical literature [4].

EES is a rare soft-tissue tumor, with an incidence rate of 0.4 per million, which is 10 times lower than the incidence of ESB. It presents as a rapidly growing soft-tissue mass and typically causes localized pain. Common primary sites include the upper thigh, buttocks, upper arm, and shoulders. At the time of diagnosis, approximately 25% of patients have metastases, which most frequently involve the lungs, bones, and bone marrow. Consequently, the symptoms experienced by the patient are dictated by the primary tumor site as well as the location of any metastases [4].

Khan et al.'s study on ES demographics and incidence found that the disease primarily affects individuals under 20 years of age and is more prevalent in men. Furthermore, the extremities are reported as a more common site for the disease compared to other body parts [5].

In the current case, the patient was a 14-year-old female with typical symptoms of hoarseness of voice, without dyspnea or dysphagia. Based on the initial diagnosis with a FOL, laryngeal cancer was suspected.

Diagnosing ES can be challenging in young patients, often leading to delays in seeking primary care. This is because children may struggle to articulate their symptoms, which are frequently misattributed to more common issues like trauma or infection. Therefore, if a child or adolescent between the ages of 12 and 20 presents to a general physician with non-specific complaints such as pain or regional swelling, it is crucial to exclude ES before initiating other treatment pathways [5].

ESFT is now recognized as a spectrum of small, round cell entities, previously classified separately based on their differentiation. Historically, before the routine use of molecular and cytogenetic analysis, the diagnosis of these small, round cell tumors relied on clinical, radiological, and histopathological features. These characteristic features typically include CD99 staining and the classical t(11;22)(q24;q12) translocation, both of which are present in the majority of, but not all, cases of ES and primitive neuroectodermal tumor (PNET). Crucially, the unifying, defining characteristic for all ESFT tumors is now considered to be the presence of chimeric transcripts involving the EWSR1 gene and a member of the ETS-domain transcription factor family, most commonly FLI1 [2].

The differential diagnosis for small, round, blue cell tumors encompasses a range of malignancies, including rhabdomyosarcoma, synovial sarcoma, malignant lymphoma, neuroblastoma (primary or secondary), and ES and PNET. Establishing a diagnosis of ES or PNET requires a comprehensive approach, considering morphological and IHC features, ultrasonographic findings, and genetic alterations [6]. Key IHC staining is used to differentiate ES/PNET from other tumors: To exclude lymphoblastic lymphoma and rhabdomyosarcoma, negative staining for leukocyte common antigen (LCA), CD30, myosin, actin, and myoglobin is required. To exclude neuroblastoma, negative staining for neurofilament, neuron-specific enolase (NSE), and S-100 protein is necessary. Furthermore, electron microscopy helps distinguish “classic” ES from PNET by identifying neurosecretory-type granules associated with microtubules and intermediate filaments (presumably neurofilaments) in PNET [7].

Similarly, in our case, the patient underwent FDG PET to evaluate for metastasis. An accurate report of the biopsied tissue showed small, dark, round, blue cells, consistent with those seen in ES. IHC reported CD99, NKX2.2, and FLI1 cells as positive. Ultimately, the patient was diagnosed with ES of the larynx.

Studies involving large cohorts of ES patients have explored optimal treatment strategies for disease located in extraskeletal sites and the head and neck region. These investigations consistently show the advantages of a multimodal therapeutic approach [3]. EES of the larynx typically consists of surgical resection, chemoradiotherapy, or a combined approach [2]. The most effective chemotherapy regimens for EES/PTEN include combinations of vincristine, doxorubicin, cyclophosphamide, ifosfamide, dactinomycin, and etoposide. Similarly, in our patient, we used neoadjuvant therapy with alternating VAC/IE therapy. Response rates are usually high (90%) [6].

The decision to pursue a larynx preservation approach, rather than total laryngectomy, holds immense significance, particularly for a 14-year-old patient. Beyond the critical objective of disease eradication, preserving the larynx ensures the retention of the patient's natural voice, an essential element of identity, communication, and social integration during formative adolescent years and throughout their lifetime. A total laryngectomy, while sometimes necessary for tumor control, would result in permanent voice loss, requiring the patient to rely on alternative methods of communication, which can carry a heavy psychological burden and significantly impact quality of life, self-esteem, and future career prospects. Successfully achieving a complete metabolic response with chemotherapy and radiation, as in this case, demonstrates that organ-sparing strategies are viable in select patients with laryngeal EES, prioritizing both oncologic outcome and the patient's long-term functional and psychological well-being by restoring a normal voice.

In a case report by Alhomsi et al, they treated their patient with surgery (laryngectomy) followed by chemoradiotherapy. The decision to treat the patient with chemoradiotherapy comes after discussion with the patient, who opted for organ conservation [3].

Wygoda et al. documented the case of an ES of the larynx with cartilage invasion. The patient refused surgery, and hence, they treated the patient with chemotherapy followed by radiotherapy. Overall treatment time was 6.5 months in this case report [6]. Similarly, metastasis to the larynx from various sites is also not uncommon, and careful histopathologic examination is a must [8].

In a peculiar case reported by Pasha et al., a non-metastatic ES of the larynx was documented in a five-year-old boy. Post excision of the tumor, the patient was advised chemoradiotherapy [9].

Non-surgical treatment approaches for this malignancy are also noteworthy. For instance, a case report described a 53-year-old man with metastatic ES of the larynx who did not undergo surgery. His treatment involved alternating cycles of vincristine-actinomycin-D-cyclophosphamide and etoposide-ifosfamide. One year following the completion of therapy, the patient showed no evidence of active or metastatic disease [2].

Table 1 summarizes reported cases of laryngeal ES in the literature, along with their management strategies. In the current case, the patient has been offered a multimodal therapeutic approach. The patient is tolerating the treatment well, and there is no evidence of metastasis.

**Table 1:** Review of literature of other reported cases of Ewing's sarcoma of the larynx. CTx: chemotherapy; RT: radiotherapy

Age	Sex	Symptoms	Size	Treatment	Reference
1 day	Male	Stridor	1 cm	Total laryngectomy	Abramowsky and Witt, 1983 [10]
74 years	Male	Acute respiratory distress	3.5 × 2.0 cm	Total laryngectomy	Yang and Hong, 2004 [8]
68 years	Male	Hoarseness, aphonia	2.0 × 1.9 × 1.7 cm	CTx plus RT	Wygoda et al., 2013 [6]
45 years	Female	Rapidly growing lump, hoarseness	2.9 cm	CTx plus RT	Lynch et al., 2014 [11]
33 years	Female	Hoarseness	Not reported	Microscopic resection plus CTx	Ijichi et al., 2016 [12]

## Conclusions

To the best of our knowledge, this is one of the few reported cases of primary EES of the larynx managed with chemotherapy and chemoradiation. The patient was treated with a combination of chemotherapy and radiation therapy. Given the rarity of laryngeal EES, definitive conclusions regarding tumor behavior and optimal treatment remain limited. Although our treatment approach resulted in favorable outcomes, additional case reports are needed to better define optimal management strategies. The lack of molecular confirmation of an EWSR1 rearrangement is a limitation of this case report. This case highlights the importance of early recognition, thorough metastatic workup, and a multidisciplinary treatment approach, and underscores the need for further reports to define prognostic factors and evidence-based management strategies better.
